# Small Regulatory RNAs May Sharpen Spatial Expression Patterns 

**DOI:** 10.1371/journal.pcbi.0030233

**Published:** 2007-11-30

**Authors:** Erel Levine, Peter McHale, Herbert Levine

**Affiliations:** Center for Theoretical Biological Physics, University of California San Diego, La Jolla, California, United States of America; University of Auckland, New Zealand

## Abstract

The precise establishment of gene expression patterns is a crucial step in development. Formation of a sharp boundary between high and low spatial expression domains requires a genetic mechanism that exhibits sensitivity, yet is robust to fluctuations, a demand that may not be easily achieved by morphogens alone. Recently, it has been demonstrated that small RNAs (and, in particular, microRNAs) play many roles in embryonic development. Whereas some RNAs are essential for embryogenesis, others are limited to fine-tuning a predetermined gene expression pattern. Here, we explore the possibility that small RNAs participate in sharpening a gene expression profile that was crudely established by a morphogen. To this end, we study a model in which small RNAs interact with a target gene and diffusively move from cell to cell. Though diffusion generally smoothens spatial expression patterns, we find that intercellular mobility of small RNAs is actually critical in sharpening the interface between target expression domains in a robust manner. This sharpening occurs as small RNAs diffuse into regions of low mRNA expression and eliminate target molecules therein, but cannot affect regions of high mRNA levels. We discuss the applicability of our results, as examples, to the case of leaf polarity establishment in maize and Hox patterning in the early *Drosophila* embryo. Our findings point out the functional significance of some mechanistic properties, such as mobility of small RNAs and the irreversibility of their interactions. These properties are yet to be established directly for most classes of small RNAs. An indirect yet simple experimental test of the proposed mechanism is suggested in some detail.

## Author Summary

Early embryonic development depends on robust patterning along the axes of the embryo. At the cellular level, neighboring segments are often identified via the concentrations of several gene products: the expression of such a gene may, for example, be high in the cells of one segment, and negligible in those of another. Recently, it has been suggested that small RNA molecules, such as microRNAs, may play a role in establishing a sharp boundary between two neighboring segments, but are not required for the overall patterning. Here, we investigate this possibility using a mathematical model, which assumes that small RNAs diffuse in the tissue. Surprisingly, we find that mobility of the small RNAs may generate a sharp interface in the expression profile of its target gene. We analyze the properties of the interaction between the two molecules that are required to achieve this function. An experimentally testable prediction is detailed, and two possible realizations in the fruit fly and in maize are discussed.

## Introduction

Morphogenesis proceeds by sequential divisions of a developing embryo into domains, each expressing a distinct set of genes. Each combination of genes is associated with a particular cell identity. At advanced stages of development, most genes that define cell identity are either highly expressed (“on”) or strongly inhibited (“off”) in a given cell. For example, two adjacent domains may be differentiated by high expression of some genes in one, and low expression in the other. In such cases, it is important that cells of the two populations do not intermix. Moreover, the number of cells that show intermediate levels of expression, typically found at the interface between the two sets, should be kept to a minimum. These demands are necessary in order to unambiguously define the identity of each cell. A spatial gene expression pattern that obeys these demands is said to exhibit a *sharp interface*.

A crucial step in setting the interfaces of gene expression patterns is often the establishment of a concentration gradient of molecules called morphogens. Some morphogens are transcription factors that regulate gene expression directly [1,2]. Others are ligands that bind cell-surface receptors signaling the activation of target expression [3]. Since morphogens act in a concentration-dependent manner, a morphogen gradient may be transformed into a gradient of its target messenger RNA (mRNA).

In principle, a single morphogen interacting cooperatively with its target enhancer can generate a sharp interface in the target transcription profile, by modulating the rate of its mRNA transcription as a function of the nuclear spatial coordinate [4]. This may be done, e.g., by cooperative binding to a receptor or to a promoter [5] or by zero-order ultrasensitivity [6]. As an example, in *Drosophila* early embryonic development, Hunchback transcription depends on the cooperative binding of about five Bicoid molecules [7]. An obvious limitation in this mechanism is the need for large cooperativity factors or cascades of reactions, which make it prone to fluctuations and slow to adapt [7–10]. Recently, a role for small regulatory RNAs in establishing developmental patterning has been documented in plants [11–13] and animals [14]. In particular, it has been suggested that microRNAs (miRNAs) confer accuracy to developmental gene expression programs [15]. This raises the possibility that small RNAs aid morphogen gradients in establishing sharp interfaces between “on” and “off” target-gene expression.

In this study, we formulate a mathematical model in which small regulatory RNAs help morphogens to determine cell identity by sharpening morphogen-induced expression patterns. For specificity, we assume here that the small RNA belongs to the miRNA family, and consider another class of small RNA in the Discussion. miRNAs constitute a major class of gene regulators that silence their targets by binding to target mRNAs. In metazoans, primary miRNA transcripts are transcribed and then processed both inside and outside of the nucleus to form mature transcripts approximately 21 nucleotides (nt) in length that are then loaded into the RNA-induced silencing complex (RISC) [16]. They are found in plants [17] and animals [18], including human [19], and are predicted to target a large fraction of all animal protein-coding genes [19–21]. In plants, miRNAs are known to affect morphology [11,12,22], implying that they play an important role in determining cell identity. This is underscored by the fact that the spatiotemporal accumulation of miRNAs is under tight control in plants [23], fly [14,24], and zebrafish [25].

Our model is constructed in one spatial dimension, namely along one spatial axis. Domains of gene expression are laid out along this axis, and we assume no significant variance along other, perpendicular axes. Two key ingredients of the model are a strong interaction between miRNA and mRNA, and intercellular mobility of the miRNA. Within this framework, miRNAs generate a sharp interface between those cells expressing high levels of the target mRNA and those expressing negligible levels of mRNA. We use physical arguments to understand the range of parameters in which this sharpening occurs. Our model predicts that the spatial position of the interface is precisely determined: mobile miRNAs spatially average individual cellular fluctuations without compromising the interface sharpness. We use computer simulations to show that this is also true even with low numbers of molecules. A consequence of our model is that a local change to the transcription profiles can induce a nonlocal effect on the mRNA concentration profile; we outline an experiment to detect this nonlocal property. Finally, we consider possible applications of these ideas in plants and fruit fly.

## Results

### Formulation of Model from Available Biological Evidence

Our theory comprises three central elements. First, miRNAs and their targets are taken to be transcribed in a space-dependent manner. Second, we assume that the interaction between miRNA and target irreversibly disable the target mRNA from being translated into proteins; this, for example, may be done by promoting the degradation of the target. Furthermore, the miRNA molecule itself may be consumed during this interaction. Last, we allow for the possibility that miRNAs move between cells. Before defining the model, let us review the available data regarding each of these processes.

miRNAs and their targets are often expressed in a coordinated manner [26]. Often, the regulatory network is designed to express the miRNA and its targets in a mutually exclusive fashion. For example, the expression patterns of the miRNA *miR-196* and its target Hoxb8 are largely nonoverlapping in mouse [27] and chick [28]. Similarly, the nascent transcripts of *ubx* (i.e., *ubx* transcripts still attached to the DNA) are expressed in a stripe near the center of the early embryo, whereas nascent transcripts of its regulator, *iab-4*, are simultaneously observed in nuclei posterior to this domain [29]. A recent large-scale study in *Drosophila* showed that miRNAs and their target genes are preferentially expressed in neighboring tissues [15]. Likewise, in mouse [30] and in human [31], predicted miRNA targets were found at lower levels in tissue expressing the cognate miRNA than in other tissues.

Our model assumes that the synthesis rate of the miRNA and its target are *smoothly* varying along a spatial axis, *x*. This, for example, may be the result of a common morphogen regulating (either directly or indirectly) the two species. The transcription profiles α*_μ_*(*x*) and α*_m_*(*x*) of the miRNA and its target are assumed to be largely anticorrelated.

The detailed interaction between miRNAs and their targets is currently a topic of intense investigation [32,33]. miRNAs induce the formation of a ribonucleoprotein complex (RISC). Targeting of a specific mRNA by a RISC is done via (often imperfect) base-pair complementarity to the miRNA [18]. Upon binding, protein synthesis is suppressed by either translational repression or mRNA destabilization [32,33]. Although it is likely that miRNA can go through a few cycles of mRNA binding [34], the increased endonucleolytic activity conferred by the miRNA makes it plausible that the miRNA is sometimes degraded in the process. In addition, evidence suggests that mRNAs that are translationally repressed by miRNA may be colocalized to cytoplasmic foci such as stress granules [35] or processing bodies [34–36]. Stress granules are cytoplasmic aggregates that appear under stress and sequester untranslated RNA, perhaps to protect these molecules or to regulate translation [37]. Processing bodies, which are enriched with endonucleases, are believed also to be places of mRNA degradation [38]. The two types of RNA granules are also known to interact, possibly an indication that stored RNA in stress granules may be targeted for degradation [37]. In both cases, RNA granules may sequester or degrade not only the mRNA, but also its bound miRNA. Taken together, these facts make it improbable that miRNAs act in a fully catalytic manner.

A pair of mRNA–miRNA reactions that describe a spectrum of plausible scenarios is

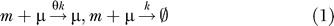
where *m* represents the mRNA concentration and *μ* represents that of the miRNA. Here, *θ* is the average number of targets degraded by a given miRNA before it is itself lost in the process. These reactions may be realized in different ways. For example, the two species may reversibly form a complex that is then subject to degradation. Another possibility is that the two species irreversibly associate to form an inert complex. Furthermore, the reaction between the species may be reversible, as long as the typical dissociation time is much longer than the relevant biological timescale. One way in which the cell may control the dissociation time is by regulating exit of the RNA pairs from processing bodies [39].

Can miRNAs move from cell to cell? Short interfering RNA (siRNAs), another important class of small RNAs, are known to elicit non–cell-autonomous RNA silencing in plants, worms, fly, and possibly mouse (reviewed in [40]). This may also be the case for *trans*-acting siRNA [13]. Evidence in favor of intercellular mobility of miRNA is found in pumpkin [41]. There, miRNAs have been found in the phloem sap that is transported throughout the plant by phloem tissue. In animals, many small RNAs, including many miRNAs, were found in exosomes from mouse and human mast cell lines, which can be delivered between cells [42].

In our model, we consider the possibility that miRNAs migrate from cell to cell. Mobility of the miRNA species is likely to rely on active export from the cell followed by import to neighboring cells, or perhaps on transport between neighboring cells, e.g., via gap junctions. On the tissue scale, these transport processes are expected to result in effective diffusion. We therefore ignore the small-scale transport processes, and model miRNA mobility as pure diffusion. Finally, we combine these processes into a steady-state mean-field model given by

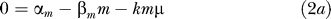


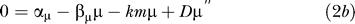



The *β* terms describe independent degradation (i.e., by processes independent of the other RNA species) and the *k* term describes coupled degradation of both RNA species. Note that the case *θ* > 0 (where miRNA may go through multiple rounds of interactions with target mRNAs) can also be brought into this form by rescaling Equation 2a [43]. Mobility of the miRNA is described by an effective diffusion constant *D*. The spatial coordinate *x* measures distance along one dimension of a tissue. All our numerical results shall be presented in units of the tissue size, i.e., 0 ≤ *x* ≤ 1.

Equation 2a and Equation 2b cannot be solved analytically. In what follows, we solve these equations numerically, imposing zero-flux boundary conditions. These exact numerical solutions can be used to draw the steady-state expression profiles of both RNA species for a particular set of parameters. To gain further insight, we also develop an approximate analytical solution.

### microRNAs May Sharpen Domain Boundaries

As described above, a desired target protein profile comprises a domain of cells that express this protein abundantly, adjacent to a domain of cells where this protein does not accumulate. Furthermore, one requires that the number of cells with intermediate expression levels lying in between the two domains be minimized—this is our definition of a sharp interface. In this section, we discuss one scenario in which the mutual consumption of a diffusive miRNA and its target leads to such a sharp interface in the mRNA profile.

**Figure 1:**
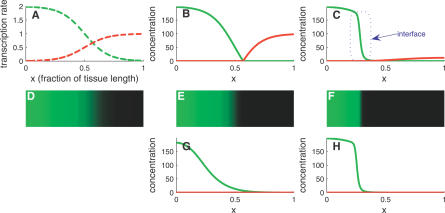
Sharpening the Target Expression Pattern (A) Transcription profiles of a miRNA (red) and its target (green). Functional expressions and parameter values are given in Methods, unless otherwise noted. (B) Steady-state concentration of target mRNA (green) and cognate miRNA (red). Regulation by immobile miRNAs (in which the diffusion constant is *D* = 0) removes residual mRNAs from the right domain, creating a sharp boundary near the center of the tissue. Here, the miRNA–mRNA interaction parameter is *k* = 1. (C) Mobility of miRNA (*D* = 0.01) further sharpens this boundary by inducing an interface between domains of gene expression (blue box). (D–F) Steady-state concentration of mRNA (green level) for a two-dimensional generalization of the model, in which diffusion occurs equally in both directions and transcription rates do not vary along the vertical axis. Parameter values of (D–F) correspond, respectively, to those in (A–C). (G) Strong miRNA diffusion (*D* increased 100-fold) smoothens the interface between the domains, but does not affect the interface position. (H) The interface structure is unaffected by increasing both the miRNA-mRNA interaction *k* and the diffusion constant *D* 100-fold with respect to (C) and (F).

We assume that some morphogen controls the transcription rate of the target. The transcription profile—namely, the transcription rate as a function of the spatial coordinate of the nucleus—is laid down as a smooth gradient, falling from one end of the developing tissue (which, for convenience, will be called “left”) toward the other end (“right”). Motivated by a recent study that showed that miRNA and their targets are preferentially expressed in neighboring tissues [15], we focus on the scenario in which the miRNA transcription is controlled in a fashion opposite to that of the mRNA: miRNA transcription is peaked at one end of the tissue (where the mRNA transcription rate is minimal), and decreases toward the other end (where the mRNA transcription rate is maximal). The kind of “mutually exclusive” transcription profiles we have in mind is depicted in Figure 1A. In this figure, and hereafter, we denote the mRNA transcription profile by α*_m_*(*x*) and the miRNA transcription profile by α*_μ_*(*x*), explicitly noting their dependence on the spatial coordinate *x*. We note that, in the absence of miRNA–target interaction and of miRNA diffusion, the concentration profiles of mRNA and miRNA simply follow their transcription profiles (Figure 1A and 1D). Each is rather smooth and overlaps the other near the center of the tissue.

Before studying the full model, it is instructive to consider first the interacting system in the absence of miRNA diffusion. In the context of mutually exclusive transcription profiles, we expect that each cell would be dominated by one RNA species (either the miRNA or the target mRNA), which we will call the majority species, and be depleted of the other, the minority species. In other words, we are making the critical assumption that the decay of the minority species in each cell is governed by the interaction with the other species (rather than by its independent degradation). This assumption can be made quantitative in terms of the model parameters; see Equation S1 in Text S1. Under this assumption, which we refer to as the *strong interaction limit*, it is straightforward to show (Equation S3 in Text S1) that the density of the majority species in each cell is proportional to the difference between the two transcription rates in that cell, whereas the minority species is essentially absent. Consequently, in the context of mutually exclusive transcription profiles, the mRNA level becomes vanishingly small in any cell for which α*_m_*(*x*) < α*_μ_*(*x*), namely every cell to the right of the point where the two transcription profiles are equal. The concentration profiles of the two RNA species are shown in Figure 1B and 1E, in which one can see that the mRNA and miRNA spatial expression domains are now complementary and more sharply defined.

The *threshold response* that arises when both RNA species do not diffuse from cell to cell provides insurance against the possibility that the mRNA transcription profile is not as step-like as is required for unambiguous cell differentiation. In other words, miRNA regulation acts as a failsafe mechanism whereby incorrectly transcribed low-abundance transcripts in the region α*_m_*(*x*) < α*_μ_*(*x*) are silenced, while correctly transcribed high-abundance transcripts in the region α*_m_*(*x*) > α*_μ_*(*x*) are only mildly affected [15,26]. This threshold response in the target profile has been observed in the context of small RNAs—another class of posttranscriptional regulators—in bacteria [43].

We now return to our full model, which allows for diffusion of the miRNA. To simplify the analysis, let us keep the strong-interaction limit, described above (and in Equation S1 in Text S1). In general, one expects that diffusion makes the miRNA profile more homogeneous, and this is confirmed by exact numerical solution of the model, as shown in Figure 1C. Surprisingly, however, the mRNA profile does not become smoother. In fact, Figures 1C and 1F show that this profile actually develops a sharper drop from high to low mRNA levels than there was in the absence of diffusion. More specifically, miRNA diffusion creates an interface between high and negligible target expression. Increasing diffusion moves the interface deeper into the mRNA-rich region and thereby accentuates the drop in mRNA levels across the interface. Although some miRNA diffusion is required to establish a sharp interface in the mRNA profile, the diffusion constant cannot be too large. As Figure 1G demonstrates, increasing the diffusion constant may result in smoothing the interface. A corresponding increase in the interaction strength, *k*, can compensate for the increased diffusion, regaining the interface sharpness (Figure 1H). We will quantify these observations below.

Diffusing miRNAs can find themselves in one of two very different regions. In the miRNA-rich region (including the region to the right of the point where the transcription profiles are equal), miRNA decay occurs mainly via processes independent of their interaction with the target. In this region, our model boils down to a simple diffusion process accompanied by linear decay. Such processes are characterized by a length scale, denoted by λ, which essentially measures how far a miRNA can travel (due to diffusion) before being consumed (by independent degradation). It is thus an increasing function of the diffusion constant *D*, but a decreasing function of the independent decay rate β*_μ_*. On the other hand, in the mRNA-rich region, a miRNA decays mainly via co-degradation with its target. In this region, miRNAs decay faster, and one expects them to be able to travel over much shorter distances than in the miRNA-rich region. In fact, diffusion in this region is characterized by another, smaller, length scale, denoted by ℓ, which again increases with *D*, but is now a decreasing function of the interaction strength, *k*. Explicit expressions for the two length scales are given in Text S1 (Equations S5 and S6 in Text S1).

To obtain a sharp interface in the mRNA profile, miRNAs should be able to travel from the miRNA-rich zone into the mRNA-rich zone. This means that the first length scale, λ, should be of the same order as the tissue length. This, for example, can be achieved if the diffusion constant *D* is large enough. On the other hand, the vicinity of the interface is governed by the other length scale, ℓ. This length scale is what determines the “width” of the interface, namely the number of cells that exhibit intermediate levels of mRNA expression (see blue box in Figure 1C). A sharp interface, therefore, means a small value of ℓ, and one way to achieve a small value of ℓ is to make the diffusion constant *D* small enough. These two contradicting requirements on *D* suggest that there might be an intermediate range of values for the diffusion constant that allows for a sharp interface, but also raises the suspicion that this range may be very small and requires some fine-tuning. This, however, is not the case: the fact that λ is strongly dependent on β*_μ_* (whereas ℓ does not depend on β*_μ_* at all), and that ℓ strongly depends on *k* (whereas λ does not) means that the range of allowed values of *D* can be set as large as needed.

In Text S1, we develop an approximate analytical expression for the mRNA profile in terms of the various parameters and the “input” profiles α*_m_*(*x*) and α*_μ_*(*x*) (Figure S1). There are two lessons to be learned from this exercise. First, the interface established by the mRNA–miRNA interaction is effectively impermeable to miRNA diffusion in the strong-interaction limit. The system thus separates into two parts which—in steady state—do not exchange molecules between them. This property allows one to calculate the position of the sharp interface in the mRNA profile.

The second lesson comes from the resulting equation for the interface position. This equation takes the form of a weighted spatial average of the difference between the two transcription profiles (Equation S11 in Text S1). Before interpreting the full result, it is instructive to consider the limiting case in which miRNAs cannot be degraded independently (β*_μ_* = 0). In this case, our result (Equation S12 in Text S1) implies that the interface is positioned such that total synthesis rates of mRNA and miRNA to its right are equal. Thus, it is the total production rates in that part of the system that determine the interface position, and not any particular cell by itself.

In the more general case (β*_μ_* > 0), the contribution of each cell is weighted by some nontrivial function. Still, in order to determine the interface position, one needs to perform a sum over many nuclei, each contributing the difference between the local transcription rates of the two RNA species. Clearly, these rates may be influenced by many factors, and in a description that is somewhat closer to reality, one would expect this difference to be fluctuating around α*_μ_*(*x*) − α*_m_*(*x*). However, the interface position is a sum of these fluctuating objects, and one might hope that the sum of these fluctuations—which are uncorrelated—would be close to zero. In this case, the interface position would be robust to fluctuations of this type. Indeed, a stochastic simulation of the model shows no change in the interface position (or structure), as compared with the deterministic model discussed so far (Figure S3; see Text S1 for details of our simulations).

In a multicellular tissue, mRNA are typically not expected to be transferred from cell to cell. Therefore, most of the work presented here does not consider the possibility that mRNA can also be mobile. Nevertheless, mRNA mobility should be considered in some cases. For example, the early *Drosophila* embryo is a syncytial blastoderm, in which nuclei multiply in a common cytoplasmic space. In the absence of cell membranes, mRNA is likely to be mobile, although probably with a small diffusion constant [44]. In Text S1, we generalize our model to include mRNA mobility. We find that a sharp interface can be achieved as long as the typical distance traveled by target mRNAs, even in the absence of miRNAs, is small compared with any other length scale (such as the interface width). Denoting the mRNA diffusion constant by *D_m_*, this condition can be written as 
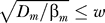
 . We note that this condition does not contradict any of the conditions mentioned before; see Figure S2.

In passing, let us note that the conditions required so far—namely, strong interaction between the miRNA and its target, and small ℓ—may be reached by making the mRNA completely stable (β*_m_* → 0). However, our analysis shows that in this case, the system would never relax to a steady state, since target mRNAs would accumulate at the left end of the tissue without limit. Our analysis here is, therefore, only applicable if the mRNA molecules undergo independent degradation, in addition to the miRNA-dependent degradation.

### microRNAs Can Define Sharp Stripes of Gene Expression

**Figure 2:**
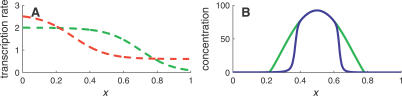
Stripe of Gene Expression Defined by microRNA Interaction (A) Transcription profiles of a miRNA (red) and its target (green), favorable for stripe generation. See Methods for parameter values. (B) Expression of the target is restricted to the middle of the tissue even when the miRNA cannot diffuse (*D* = 0, green). Mobility of the miRNA makes target expression more focused (*D* = 0.001, blue): target expression in the stripe is enhanced, and the stripe boundaries become sharp.

Using the insight gained in the previous section, we briefly show how a stripe is formed when the miRNA transcription profile α*_μ_*(*x*) is similar to α*_m_*(*x*) but displaced from it (Figure 2A). Suppose, for example, that the synthesis of an miRNA and its target are activated by the same transcription factor. In any given nucleus, the two promoters experience the same concentration of this transcription factor. However, they need not react in the same way: if the binding affinity of one promoter is stronger than that of the other, there will be intermediate concentrations of the transcription factor such that the first promoter will be activated while the other will not. Such a scenario is depicted in Figure 2A, where a common transcription factor, which exhibits a spatial gradient, activates the target gene as well as the miRNA gene. In this case, the target promoter has higher affinity to the transcription factor than the miRNA promoter. Thus, some cells in the middle of the developing tissue express the target mRNA, but not the miRNA.

Unlike the case studied in the previous section, in which the transcription profiles crossed at one point, here the transcription profiles cross at two points. Let us retrace our steps in the previous section by first considering the case of no diffusion. For low values of the interaction rate *k*, the miRNA and mRNA profiles are qualitatively similar to their transcription profiles. As *k* is increased however, miRNA deplete mRNA levels at any position where α*_μ_* > α*_m_* and thus confine mRNA expression to a stripe between the two crossing points of the transcription profiles (green curve in Figure 2B). Can diffusion make this profile sharper, as in the previous case? Indeed, diffusing miRNAs that survive annihilation on the left and right diffuse into the interval between the two crossing points, and establish sharp interfaces in the mRNA concentration profile. The resulting stripe resides within this interval, but is narrower (blue curve in Figure 2B). It is therefore important that parameters allow for sharp interfaces, without making the stripe too narrow (or even disappear). Therefore, to sustain a well-defined stripe of gene expression, the interface width must be much smaller than the distance between the two crossing points of the transcription profiles.

One can use the same analytic method mentioned earlier to calculate the new positions of the stripe boundaries (see Figure S4 and Text S1). This exemplifies how the method can be used to analyze geometries of increasing complexity.

### Standard Experimental Methods May Be Used to Probe the Sharpening Mechanism

The sharp interface that we predict can be detected directly in an imaging experiment, provided the light intensity varies linearly with mRNA concentration and the spatial resolution is high enough. However, experiments often do not supply quantitative data that are faithful to the underlying concentration profile. This, for example, is the case if an experimental setup is designed to identify the presence/absence of a molecular species. The application of nonlinear filters, such as photomultipliers, may result in spurious sharp boundaries. In contrast, low spatial resolution may make a sharp interface appear smoother than it really is.

**Figure 3:**
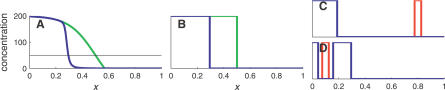
Indirect Test of the Sharpening Mechanism (A) Concentration profiles without diffusion of miRNA (green) and with diffusion (blue). The black line denotes an apparatus-dependent threshold concentration. (B) The readout from an apparatus that amplifies any signal above its detection threshold does not distinguish a smooth drop in mRNA levels (the underlying profile of the green curve) from a sharp drop (blue). (C) A patch (red) in the miRNA-rich region (right of the interface) pushes the interface to the left in a threshold assay. (D) A patch in the mRNA-rich region (left of the interface) leaves the interface unaffected. See Methods for parameter values.

Here, we address the task of making predictions that are based on quantitative analysis, yet can be tested using qualitative data. To this end, we consider a worst-case (if somewhat artificial) scenario in which the apparatus' readout is binary: concentrations below an apparatus-dependent threshold are not detected, whereas concentrations larger than this elicit a concentration-independent fluorescence intensity. In such scenarios, it is impossible to tell a smooth and sharp concentration profile apart as both yield a sharp interface in the binary readout (Figure 3A and 3B).

Fortunately, our model of miRNA-mediated morphogenic regulation possesses another signature that is visible at such coarse experimental resolution. To detect this signature, one needs to overexpress the miRNA in a small patch of cells (hereafter denoted the “patch”). Our model then predicts that this patch has a qualitatively different effect depending on which side of the interface it occurs.

The technique one uses to generate the patch may differ according to the stage of development under consideration. In the early blastoderm stages of *Drosophila* development, for example, a Gal4 driver may be used to drive expression of the miRNA in those cells in which an endogenous gene is expressed [45]. Many endogenous genes are expressed in stripes along the anterior–posterior axis during these stages and some have dedicated enhancers for single stripes [4,46]. As an example, the yeast FLP-FRT recombination system has been used to misexpress the gap gene *knirps* in a stripe by placing it under the control of the *eve* stripe 2 enhancer [47].

In later stages of *Drosophila* development, e.g., imaginal discs, one technique is the random generation of a mosaic of mutant clones (patches) by mitotic recombination [45,48]. The patches are generated at a low rate, and one then screens for those embryos containing a single patch.

We model the localized overexpression of miRNA by an effective local increase of the transcription rate (by an amount α*_c_* = 5). This increase in transcription rate occurs in a small number of cells, which in our model is about 5% of the tissue length. More specifically, we choose a position *x_c_* for the center of the patch, and for every point *x* that resides within a distance *w*/2 of *x_c_*, we change the transcription rate from α*_μ_*(*x*) to α*_μ_*(*x*) + α*_c_*. Here *w* is the “width” of the patch, which takes a value *w* ≃ 5% of the tissue length.

Consider positioning the patch first in the miRNA-rich region of the developing tissue (Figure 3C). One sees that, even if positioned at a distance from the expression domain of the target, the effect of the additional miRNA is to push the interface toward the left. The localized patch of cells therefore has a nonlocal effect.

As mentioned earlier, the position of the interface is determined by a global comparison of the mRNA and miRNA transcription rates to the right of the interface. Ectopic expression of miRNA to the right of the interface changes this balance and displaces the boundary. Note that this displacement can only be achieved if the patch is positioned to the right of the interface, since the interface position is not influenced by transcription balance to its left. This effect is quantified in Text S1.

This experiment should be contrasted with one in which the overexpressing patch is positioned in the mRNA-rich region, as shown in Figure 3D. Such ectopic miRNA expression has a local effect, with excess miRNA creating a hole in the mRNA expression domain. The hole edges constitute two additional interfaces in the system, the sharpness of each again determined by ℓ.

**Figure 4:**
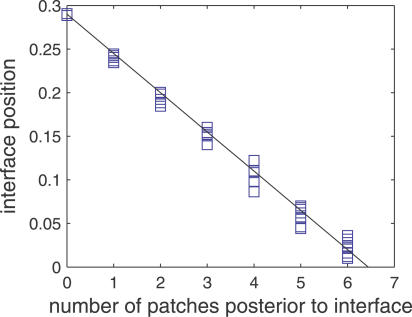
Quantitative Test of the Sharpening Mechanism Simulated experiment in which the interface position *x_t_* is measured in an ensemble of samples having various numbers of patches. The theory predicts that the interface displacement should be linearly proportional to the number of patches. Parameter values are as in Figure 3.

One can go further and make a quantitative prediction, relating the number of patches in a mosaic of patches with the lateral shift in the interface position. To first approximation, one needs to count the number of patches in the miRNA-rich region, and disregard completely the patches in the mRNA-rich region. The displacement of the interface position is then linearly proportional to this number; see Text S1 for details. Simulated experimental results that would verify this prediction are shown in Figure 4.

The distinct nonlocal effect described above does not occur when the miRNA are unable to move between cells. Also, we have checked (for the parameters used in this study) that it does not occur when the miRNA acts purely catalytically (Figure S5). Rather, both miRNA mobility and a strong interaction between miRNA and target are required. The presence or absence of the nonlocal effect would therefore confirm or falsify the hypotheses that miRNA are mobile and that they interact stoichiometrically with mRNA while in this mobile state.

## Discussion

In this study, we have analyzed a model in which miRNAs sharpen target-gene expression patterns by generating an interface between high and low target expression. This effect is due only to the strong noncatalytic interaction between the miRNA and its target, and requires no additional interactions or feedbacks.

A necessary condition for a sharp interface to occur is that the miRNA and target are co-degraded; a miRNA–mRNA interaction in which miRNAs promote mRNA degradation, but in which miRNAs themselves are unaffected, is insufficient. One can, in principle, test the existence of coupled miRNA–mRNA degradation by inhibiting the transcription of mRNA and monitoring its decay rate, which in this case would be time-dependent, 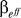 (*t*) = β*_m_* + *k μ*(*t*). We note also that a stoichiometric interaction may complicate the interpretation of sensor transgene experiments [27], as the transgene would then sequester miRNA and thereby alter the original expression patterns.

In principle, any interaction between a pair of molecules that obeys the rules of our model, such as an irreversible noncatalytic interaction, can set up a sharp expression interface. For example, suppose that a transcription factor is deactivated by binding irreversibly to an inhibitor protein. In this case, the concentration profile of active transcription factors can exhibit a sharp interface via the mechanism described above. In common with classical reaction–diffusion models for developmental patterning [49,50], an essential property here is that the inhibitor diffuses much faster than its target.

The interface between low and high mRNA levels is characterized by low molecule numbers of both RNA species. In such cases, fluctuations in the molecule number of either species may have macroscopic effects. For example, a small RNA-target pair in bacteria shows enhanced fluctuations when their transcription rates become comparable (E. Levine, M. Huang, Y. Huang, T. Kuhlman, Z. Zhang, and T. Hwa, unpublished data) [51]. These fluctuations can in turn give rise to noise-induced bistability, which manifests itself experimentally as diversity in a population of cells (E. Levine, M. Huang, Y. Huang, T. Kuhlman, Z. Zhang, and T. Hwa, unpublished data). We performed Monte Carlo simulations of our model, but found that fluctuations have no macroscopic effect, even near the transition point where molecule numbers of both species are low. This in-built robustness to fluctuations arises because the interface position is determined by an integrated transcriptional flux which averages out individual cellular fluxes. Thus spatial averaging results in high spatial precision without smoothing out the interface.

Strong cooperative activation, as often occurs in morphogenetic regulation at the transcriptional level (e.g., Bicoid has about five binding sites in target promoters of *Drosophila*), would seem to make pattern formation by morphogens inherently susceptible to temperature variations [52,53]. Nevertheless, embryonic patterning appears to be quite robust to temperature variations, as has been documented for Hunchback [52] and for Eve [54] in *Drosophila*. The only cooperative reaction required in the model presented in this work is coupled degradation of miRNA and mRNA, suggesting the possibility that miRNAs filter fluctuations arising from temperature variations.

Candidate systems in which to test the ideas put forth in this study include the establishment of dorsoventral (adaxial/abaxial) leaf polarity in plants, as well as the segmentation of the early *Drosophila* embryo. We now discuss these two systems in some detail.

Leaf polarity in plants is established shortly after the emergence of the leaf primordium from the meristem. Specification of leaf polarity depends on the Sussex signal [55], a meristem-borne signal that specifies adaxial cell fates. Members of class III of the homeodomain-leucine zipper (HD-ZIPIII) proteins specify adaxial fate [11,56]. In *Arabidopsis* and in maize, the polar expression pattern of these genes results from their inhibition by two miRNAs, miR165/166, which exhibit a complementary expression pattern [11,12]. Recently, it has been shown that in maize, restriction of miR165/166 to the abaxial side of the developing leaf depends on the polarized expression of LBL-1, a protein involved in the biosynthesis of *trans*-acting RNAs, ta-siR2141/2 [13]. Possible targets of ta-siR2141/2 include members of the *arf3* gene family (a transcription factor that is expressed abaxially), as well as members of the miR166 family [13]. Although miRNAs in plants are thought to act mainly cell autonomously [57], DCL4-dependent siRNAs, such as ta-siR2141/2, may exhibit cell-to-cell movement [40].

**Figure 5:**
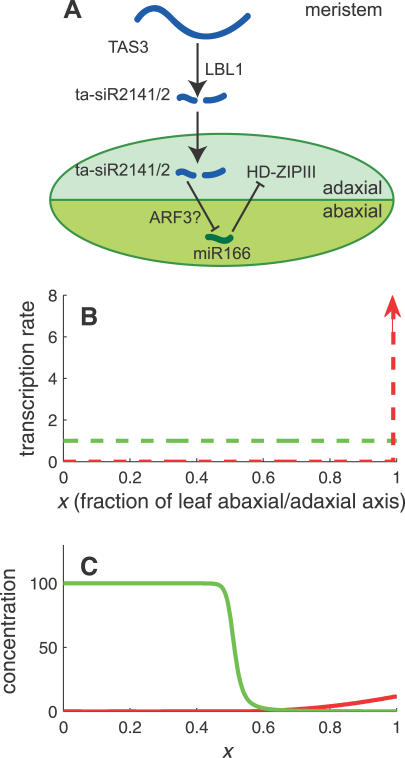
Model for Leaf Polarity in Maize (A) In this model, TAS3 is processed in the meristem into several ta-siRNAs. Two of those, ta-siR2141/2, migrate into the adaxial side of the primordial leaf, where they inhibit miR166 (either directly or via ARF3). miR166 goes on to repress expression of HD-ZIPIII genes, which define adaxial fate. (B) Transcription profiles. Transcription of ta-siR2141/2 (red) occurs in the shoot meristem, next to the adaxial side of the developing leaf. The target, either miR166 or arf3, is transcribed uniformly throughout the leaf (green). (C) Steady-state concentrations of ta-siR2141/2 (red) and its target (green). Diffusion of ta-siR2141/2 into the leaf restricts the expression of the target to the abaxial side, and—assuming a noncatalytic interaction—creates a sharp interface between the abaxial and adaxial domains. See Methods for parameter values.

The following model is consistent with these data (Figure 5A). The RNA transcript TAS3 is cleaved to produce ta-siR2141/2 in the meristem. These small RNAs then propagate (diffuse) into the adaxial side of the leaf, inhibiting the expression of miR166 either directly or through the ARF3 transcription factor. The target (either miR166 or ARF3) is transcribed uniformly throughout the leaf, and is localized to the cell where it is synthesized. If one further assumes that the interaction between ta-siR2141/2 and its target is noncatalytic, then this model belongs to the class of models studied in this work, and can therefore exhibit a sharp interface between the abaxial domain of high target expression and the adaxial domain of no expression; see Figure 5. In agreement with this model is the low abundance of ta-siR2141/2 in *Arabidopsis* [58,59], despite their distinct phenotypic role.

Early embryonic development in *Drosophila* proceeds via a cascade of gene activities that progressively refine expression patterns along the anterior–posterior axis of the embryo. A recent study of the expression patterns of nascent miRNA transcripts suggests that a number of miRNAs play a role in this process. The miRNAs *miR-309clus*, *miR-10*, and *iab-4* (which all reside between annotated mRNA genes on the genome), and *miR-11*, *miR-274*, and *miR-281clus* (which all reside within introns of annotated genes) are all expressed in a graded fashion along the anterior–posterior axis of the blastoderm embryo [14,60].

The complementary transcription profiles of *iab-4* and its target *ubx* at stage 5 of development make this miRNA-target system a candidate for the sharpening mechanism proposed in this study. The early *ubx* transcript pattern is broadly distributed over the posterior half of the embryo, becoming localized to a stripe at the center of the embryo by the completion of cell formation [29,61], probably as a result of transcriptional repression by Hunchback in the anterior and posterior regions of the embryo [62]. The nascent transcript profile of its regulator, *iab-4*, is broadly distributed posterior to this stripe [29].

**Figure 6:**
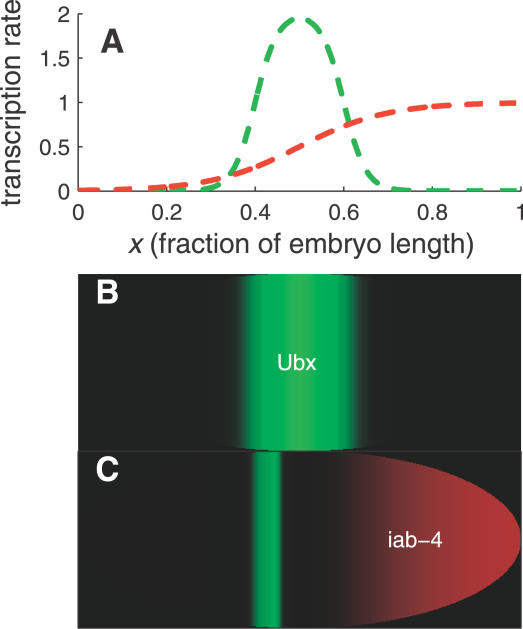
Model for Sharpening of *ubx* Expression Boundaries by *iab-4* (A) The assumed transcription profiles of *ubx* (green) and *iab-4* (red). (B) In the absence of *iab-4* (or when the interaction between *iab-4* and *ubx* is inhibited), the expression pattern of *ubx* is broad and smooth. (C) When *iab-4* and *ubx* interact, the expression pattern of *ubx* becomes narrow and sharp. The diffusion constant is *D* = 0.001; see Methods for other parameter values. The embryo-like shape is for illustrative purpose only.

It may be the case that *iab-4* is also expressed before cell formation and that the absence of cell membranes makes *iab-4* mobile. The much larger *ubx* mRNA, on the other hand, may be effectively stationary on the timescales of interest [7]. Furthermore, the transcription profiles of *iab-4* and *ubx* at stage 5 do not seem to overlap [29], suggesting that *iab-4* intercellular mobility is crucial to allow it to interact with *ubx* at this stage of development. Assuming then that only the miRNA *iab-4* is mobile, the complementary expression patterns of *iab-4* and its target, *ubx*, measured in [29] is consistent with our model of miRNA-induced sharpening. Sample profiles predicted by the model are shown in Figure 6.

A possible difficulty with regard to applying our model to *ubx*/*iab-4* is that the system may not have reached steady state before stage 6, when cells begin to migrate. In particular, no *ubx* protein was detected at stage 5, possibly because of the time needed to transcribe the large *ubx* locus [29].

Like *iab-4*, the miRNA *miR-10* is also expressed at stage 5 in a broad posterior region along the anterior–posterior axis [14]. The homeotic gene *Scr* is a predicted target of miR-10 [63] and is also expressed in the blastoderm at stage 5 [64]. The miR-10 site in the *Scr* 3′ UTR is likely to be functional because the pairing is well conserved in all drosophilid genomes and because the miRNA site is conserved in the *Scr* genes in mosquito, the flour beetle, and the silk moth [20]. Unlike *ubx*, the protein of Scr is detected at this stage of development in a stripe of ectodermal cells about four cells wide in the parasegment-2 region, though it may not be functional at this time as the protein (a transcription factor) was not localized to the nucleus [64]. This spatial expression pattern is proximal to the anterior limit of *miR-10* expression [14,64]. Hence the interaction of miR-10 with Scr at stage 5 of *Drosophila* development is also a candidate for the sharpening mechanism.

The sharpening mechanism is most effective when the spatial transcription profiles of miRNA and target are regulated in such a way as to be mutually exclusive. The genomic locations of the miRNAs *iab-4* and *miR-10* are proximal to their targets, which is certainly consistent with the possibility of coordinated regulation [65].

## Methods

To obtain the concentration profiles for the mRNA and miRNA in the different scenarios considered in this paper, we integrated numerically Equation 2. To do this, one needs to specify the transcription profiles, α*_m_*(*x*) and α*_μ_*(*x*), and the values of the parameters β*_m_*, β*_μ_*, *k*, and *D*. Unless mentioned otherwise in the text, we chose β*_m_* = β*_μ_* = *D* = 0.01 and *k* = 1 throughout.

The transcription profiles of Figures 1 and 3 were

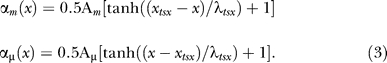
where *A_m_* = 2, *A_μ_* = 1, *x_tsx_* = 0.5, and λ*_tsx_* = 0.2. In the stripe geometry (Figure 2), the transcription profile for *m* was as above, with *x_tsx_* = 0.7. The transcription profile of the miRNA was given by

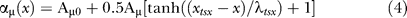
with *A_μ_* = 2, *A_μ0_* = 0.6, and *x_tsx_* = 0.3.

In the Discussion, we outline possible applications in two systems: leaf polarity in maize and segmentation in the early *Drosophila* embryo. Here, we did not aim to estimate parameters from experimental data (which, in most cases, is not quantitative enough to allow for parameter inference). Instead, parameters were chosen arbitrarily to allow clear demonstration of possible results. In the case of leaf polarity (Figure 5), we chose α*_m_*(*x*) = *A_m_*, α*_s_*(*x*) = *A_μ_*θ(*x* − *x_tsx_*) with *A_m_* = 1,*A_μ_* = 50, and *x_tsx_* = 0.99. Here, θ(*x*) is the unit step function. In the *Drosophila* embryo (Figure 6), the transcription profile of the *iab-4* miRNA was the same as in Equation 3, whereas the *ubx* mRNA transcription profile was given by

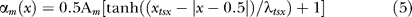
with *x_tsx_* = 0.1, *λ_tsx_* = 0.05, and *A_m_* = 2.

## Supporting Information

Figure S1 Analytical Approximation for a Sharp Boundary A comparison of the exact numerical concentration profiles with the analytical approximation (grey lines) described in the text. Note that the interface is always sharp in the analytic approximation. The diffusion constant here is *D* = 0.01, and the miRNA–mRNA interaction parameter is *k* = 1. Independent degradation rates are β = 0.01.
(17 KB EPS)

Figure S2 Sharp Interface in mRNA Expression Profile Can Be Generated Even in the Presence of mRNA Diffusion mRNA diffusion is characterized by a diffusion constant *D_m_*. Here, we compare the case of no diffusion (*D_m_* = 0, green), as in Figure 1C, with cases in which *D_m_* = *D*/100 (blue) or *D_m_* = *D*/1000 (magenta).
(9 KB EPS)

Figure S3 Stochastic Simulations Confirm Mean-Field Model Temporal averages (squares) of a stochastic simulation of the model, compared with the corresponding mean-field solution (solid lines), in a developing field comprising 100 cells. The diffusion constant is *D* = 1,000, and the miRNA–mRNA interaction parameter is *k* = 100. The autonomous degradation rates are β = 0.1.
(43 KB EPS)

Figure S4 Analytical Approximation for a Stripe Geometry A comparison of the exact numerical concentration profiles with the analytical approximation (grey lines) described in the text when the transcription profiles are aligned so as to produce a stripe. See caption of Figure S1 for parameter values.
(17 KB EPS)

Figure S5 Catalytic Interaction between miRNA and Its Target Does Not Generate a Sharp Interface To model a case in which miRNAs act catalytically, the *k*-term in Equation 2b is dropped.
(A) Here, we use parameters as in Figure 1C, except that miRNA expression is reduced 100-fold. Although noncatalytic interactions generate a sharp interface in this case, catalytic interactions make it smoother (compare the mRNA profile here and its transcription profile in Figure 1A).
(B) In the catalytic case, ectopic expression of miRNA in a patch to the right of the interface does not shift the position of the mRNA profile. Parameters here are the same as in Figure 3C.
(14 KB EPS)

Text S1 Details of Models and Calculations (52 KB PDF)

### Accession Numbers

The GenBank (http://www.ncbi.nlm.nih.gov/Genbank/) GeneIDs for the genes discussed in this paper are *arf3* (817014), *eve* (36039), *hb* (41032), *hoxb8* (15416), *iab-4* (3772110), *lbl1* (100037819), *miR-10* (3772568), *miR-11* (3771987), *miR-196* (387191), *miR-274* (3771876), *miR-281–1* (3772402), *miR-281–2* (3772497), *miR-309* (3772613), *scr* (40833), *tas3* (3768766), and *ubx* (42034).

## Abbreviations

miRNA: microRNA
siRNA: short interfering RNA
