# Supplementary material for: Small Regulatory RNAs May Sharpen Spatial Expression Patterns
Source: PLoS Comput Biol. 2007 Nov 30;3(11):e233. doi: 10.1371/journal.pcbi.0030233 (PMC2098861; doi:10.1371/journal.pcbi.0030233)
Supplement: Text S1 — (52 KB PDF) [file pcbi.0030233.sd001.pdf]

SUPPORTING INFORMATION

Strong-interaction limit

Here we define, for the case of no diffusion, what we mean by the strong-interaction limit, but the limit is equally valid when diffusion is present. We make the critical assumption that at any cellular position the decay of the minority species is dominated by coupled degradation; by rescaling Eq. 2 of the main text, this condition amounts to

$$\max\left\{\frac{k\alpha_m(x)/\beta_m}{\beta_\mu}, \frac{k\alpha_\mu(x)/\beta_\mu}{\beta_m}\right\} \gg 1, \text{ for all } x. \quad \text{(S1)}$$

In the absence of diffusion, Eq. 2 boils down to a quadratic equation for the steady-state mRNA level m, and one can immediately write the solution

$$m = \frac{1}{2\beta_m}\left[\alpha_m - \alpha_\mu(1+\epsilon) + \sqrt{[\alpha_m + \alpha_\mu(1+\epsilon)]^2 - 4\alpha_m\alpha_\mu}\right] \quad \text{(S2)}$$

where $\epsilon \equiv \beta_m\beta_\mu/(k\alpha_\mu)$, which is assumed in (S1) to be small. To zeroth order in ϵ, this expression simplifies to

$$m \approx \frac{[\alpha_m - \alpha_\mu]_+}{\beta_m}, \quad \text{(S3)}$$

where $[x]_+ = \max\{0, x\}$, as depicted in Fig. 1B.

Analytical Approximation

To understand the origin of the length scales λ and ℓ, and their relation to the tissue length L when the interface is sharp, consider first the region of space where miRNA are in the majority. In this region, where $k\mu \gg \beta_m$, we neglect the idependent-degradation term in (2a), yielding $\alpha_m = km\mu$ and thus

$$0 = \alpha_\mu - \alpha_m - \beta_\mu\mu + D\mu'' \; . \quad \text{(S4)}$$

Hence miRNA are produced at an effective rate $\alpha_\mu - \alpha_m$ and diffuse over distances of order

$$\lambda = \sqrt{\frac{D}{\beta_\mu}} \; , \quad \text{(S5)}$$

which, as we have argued in the main text, should be comparable to the tissue length, $\lambda \sim L$. On the other hand, in the mRNA-rich zone $km \gg \beta_\mu$ and so the only length scale available to the miRNA is

$$\ell = \sqrt{\frac{D}{k\alpha_m^*/\beta_m}} \; , \quad \text{(S6)}$$

where α_m^* is a typical value of $\alpha_m(x)$ in the mRNA-rich zone. For the mRNA this is the only length scale that competes with the spatial layout provided by the transcription profile and so it must determine the interface width up to a constant prefactor p. Hence the second condition for a sharp interface is that $p\ell \ll L$. In agreement with the expression in Eq. S6, derived on heuristic grounds, our numerical solutions show that the interface becomes broader when the co-degradation rate k is decreased (Fig. 1G) or when the diffusion constant D is increased (Fig. 1H). We note that the limit $p\ell \ll L$ is equivalent to

$$k\alpha_m^*/\beta_m \gg D/L^2. \quad \text{(S7)}$$

In other words, a sharp interface arises when the co-degradation rate of miRNA and target dominates the rate of diffusion over macroscopic distances. Since we are neglecting diffusion of miRNA in the mRNA-rich region, the mRNA profile there is given again by (S3).

Microscopically, miRNA in the miRNA-rich zone diffuse in a landscape dominated by independent degradation, leading to the decay length λ in (S5). Upon entering the mRNA-rich region, co-degradation suddenly overwhelms independent degradation of miRNA ($km \gg \beta_\mu$), and the miRNA are faced with an effective absorbing boundary. We therefore expect the miRNA concentration to vanish as one approaches the interface from the right. In addition, our picture asserts that the miRNA concentration is vanishingly small everywhere on the left of the interface. Taken together, these two properties impose zero miRNA concentration and zero miRNA diffusive flux at the interface. These two boundary conditions on the miRNA dynamics at the interface between mRNA-rich and miRNA-rich regions, together with the zero-flux condition at $x = L$, allow us to determine the position x_t of the interface. Furthermore, the interface must lie in the region defined by $\alpha_m > \alpha_\mu$ because co-degradation can dominate independent degradation of miRNA only if there is a reservoir of mRNA to co-degrade with.

Armed with this insight into the miRNA profile we may now solve (S4), subject to the boundary conditions $-D\mu'(x_t) = -D\mu'(L) = 0$ and $\mu(x_t) = 0$, in terms of a Green's function. Making use of the zero-flux boundary conditions, the Green's function of (S4) is

$$g(x,s) = \begin{cases} G(x,s) & \text{if } x < s \\ G(s,x) & \text{if } x > s \end{cases} , \quad \text{(S8)}$$

where

$$\lambda\, G(x,s) = \frac{\cosh\left(\frac{x-x_t}{\lambda}\right)\cosh\left(\frac{L-s}{\lambda}\right)}{\sinh\left(\frac{L-x_t}{\lambda}\right)}. \quad \text{(S9)}$$

The miRNA profile is then a weighted spatial average of the net transcriptional flux of miRNA to the right of the interface

$$\beta_\mu\mu(x) = \int_{x_t}^{L} [\alpha_\mu(s) - \alpha_m(s)]g(x,s)\, ds. \quad \text{(S10)}$$

Employing the zero-concentration boundary condition $\mu(x_t) = 0$, we arrive at the following implicit equation for x_t

$$\int_{x_t}^{L} [\alpha_\mu(s) - \alpha_m(s)]\, g(x_t, s)\, ds = 0. \tag{S11}$$

As mentioned in the text, this equation takes a simple form in the limit $\beta_\mu \to 0$, where $g(x, s)$ becomes a constant, and one has

$$\int_{x_t}^{L} \alpha_\mu(s) ds = \int_{x_t}^{L} \alpha_m(s) ds. \tag{S12}$$

In either case, solving for x_t requires knowledge only of the transcription profiles. Moreover, one immediately sees that x_t can tolerate fluctuations in the transcription profiles which preserve the integral. This should be contrasted with the non-diffusive case in which x_t reduces to the crossing point of the transcription profiles, $\alpha_m(x_t) = \alpha_\mu(x_t)$, which is less robust to small-number fluctuations.

In Fig. S1 we compare the analytical expressions in Eqs. S3, S10 and S11 with the exact numerical solution of (2). As expected the agreement is good because co-degradation dominates both independent degradation (Eq. S1) and loss due to diffusion (Eq. S7).

Diffusion of mRNA

To address mRNA mobility, we generalize our model by replacing Eq. 2a with

$$0 = \alpha_m - \beta_m m - k m \mu + D_m m'' , \tag{S13}$$

where D_m is the mRNA diffusion constant. In Fig S2 we compare numerical solutions for the generalized model for $D_m = 0, D/100$ and $D_m = D/1000$, where D is (as before) the diffusion constant of the miRNA species. At finite but small mRNA diffusion constant a sharp interface is still observed.

Sharpening of gene expression profile can also occur via the mechanism described in the main text, provided that the distance traveled by the mRNA is short compared with other length scales, in particular the interface width w. The typical distance traveled by mRNAs in the absence of miRNAs is given by $\ell_m = \sqrt{D_m/\beta_m}$. This distance can be made sufficiently small, $\ell_m \lesssim w$, either by having a small diffusion constant D_m, or by mRNAs which are inherently unstable (large β_m). Note that reducing β_m compromises two other conditions, Eqs. S1 and S7. However, this can be compensated by a stronger miRNA-mRNA interaction (large k). To demonstrate this effect, we multiplied D_m, β_m and k by 10 with respect to the values used for the magenta curve. Plotting $\beta_m m$ for the two sets of parameters then yielded two indistinguishable curves.

Stochastic Simulations

We used the Gillespie algorithm to stochastically simulate the reaction and diffusion events on a one-dimensional grid of cells.In this algorithm the next event, as well as the time to the next event, are chosen randomly. A simulation using 100 cells (the approximate anterior-posterior length of the *Drosophila* embryo during cycle 14) is compared with the corresponding deterministic solution in Fig. S3. Surprisingly, the deterministic solution is a good approximation for mRNA anterior abundances as low as 20 molecules per cell. As expected, in cases where the predicted interface is of the order of a single cell we find that the solution to our mean-field model underestimates the width of the interface. This, for example, is the case when the developing tissue becomes as small as 10 cells—the approximate size of the leaf-organ primordium during plant development.

Stripe Boundaries

We denote the left-most boundary of the stripe by x_{t1} and the right-most by x_{t2}. The location of these interfaces is determined analytically by solving (S4) with zero-flux boundary conditions in the interval $[0, x_{t1}]$ (and then enforcing $\mu(x_{t1}) = 0$) and in the interval $[x_{t2}, L]$ (and then enforcing $\mu(x_{t2}) = 0$). In the region between the interfaces the mRNA profile is approximately given by $m = [\alpha_m - \alpha_\mu]/\beta_m$; in the portion of the developing tissue complementary to this the mRNA profile is negligible. The analytic profiles for μ and m are compared with the exact numerical solutions in Fig. S4.

Experimental Prediction

The nonlocal effect predicted when the miRNA is overexpressed in a small patch of cells can be understood with the aid of (S4). First, integrate this equation from the interface to L, both with $(\hat{x}_t)$ and without (x_t) the patch. Then, neglecting the change in the miRNA concentration induced by the patch, one can show that

$$\alpha_c w \approx \int_{\hat{x}_t}^{x_t} \alpha_m. \tag{S14}$$

In other words miRNAs from the patch diffuse across the tissue towards the region $[\hat{x}_t, x_t]$ and annihilate all mRNAs that would otherwise have maintained the interface at x_t.

As mentioned in the main text, we can make a quantitative testable prediction when there are a number of independent patches. To see this recall that Eq. S14 is an integral relationship. In the corresponding equation for multiple patches, the left-hand side is proportional to the number of patches, provided we make the reasonable assumption that the patches are uniform in size and transcription rate. Similarly, the right-hand side is proportional to the interface shift, $x_t - \hat{x}_t$, provided the mRNA transcription profile is sufficiently flat in the

interval $[\hat{x}_t, x_t]$. Hence the interface position $\hat{x}_t$ decreases linearly with the number of patches. This prediction can readily be tested using an ensemble of embryos with varying numbers of patches. Simulated experimental results that would verify this prediction are shown in Fig. 4 in the main text.
